# Plasma Irisin Modestly Increases during Moderate and High-Intensity Afternoon Exercise in Obese Females

**DOI:** 10.1371/journal.pone.0170690

**Published:** 2017-01-26

**Authors:** Nathan C. Winn, Zachary I. Grunewald, Ying Liu, Timothy D. Heden, Lauren M. Nyhoff, Jill A. Kanaley

**Affiliations:** 1 Department of Nutrition and Exercise Physiology, University of Missouri, Columbia, Missouri, United States of America; 2 East Carolina Diabetes and Obesity Institute and Department of Kinesiology, East Carolina University, Greenville, North Carolina, United States of America; East Tennessee State University, UNITED STATES

## Abstract

**Background and Purpose:**

Irisin is an exercise-responsive myokine that has been proposed to exert anti-obesity benefits; yet its response during exercise in obese women is not described. This study characterized plasma irisin levels during a single bout of afternoon isocaloric-exercise of different intensities (moderate- vs high-intensity) in obese females.

**Methods:**

Eleven obese females participated in 3 randomized study days beginning at 1600h: 1) no exercise (NoEx), 2) moderate exercise (ModEx; 55%VO_2_max) and 3) high intensity interval exercise (IntEx; 4 min (80%VO_2_max)/3 min (50% VO_2_max). Frequent blood samples were analyzed for glucose and lactate (whole-blood), and insulin, c-peptide, glucagon, and irisin (plasma) throughout 190 min of testing.

**Results:**

Plasma irisin increased above baseline during ModEx and IntEx (*P*<0.05), but not NoEx (*P*>0.05). Peak irisin levels during ModEx and IntEx exercise were 11.9± 3.4% and 12.3 ± 4.1% relative to baseline (*P*<0.05), respectively, with no differences between exercise intensities (*P*>0.05). Irisin levels remained elevated above resting for 125 minutes post-exercise during ModEx, whereas levels returned to baseline within 15 minutes post-exercise during IntEx. Similarly, no associations were found between plasma irisin levels and circulating lactate, glucose, insulin, c-peptide, or glucagon among study days (*P*>0.05). However, there was an inverse association between basal irisin and lean mass (r = -0.70, *P* = 0.01).

**Conclusion:**

A single bout of moderate and high intensity afternoon exercise induces modest increases in circulating irisin concentrations during exercise; however the regulation post-exercise appears to be dimorphic between exercise intensity in obese females. Future studies are needed to compare morning and afternoon exercise on irisin secretion.

## Introduction

It is well established that both acute and chronic exercise training induce favorable metabolic and biochemical adaptations that protect against the development of metabolic syndrome, type 2 diabetes, and cardiovascular disease [1–5], yet the molecular mechanisms are not completely understood [6]. Skeletal muscle has been recognized as an endocrine organ that plays an active role in metabolic homeostasis through its ability to communicate with multiple tissues including adipose tissue, liver, and brain [7]. Specifically, muscle contraction enhances the release of several myokines including irisin that may mediate some of the protective effects of exercise [7, 8].

Irisin, a muscle and adipose derived chemokine that originates from the proteolytic cleavage of fibronectin type III domain-containing protein 5, has been reported to activate thermogenic programs in white adipose tissue and improve glycaemia, an effect that is dependent on peroxisome proliferator-activated receptor-γ coactivator-1 α (PGC-1α) [8]. Specifically, Bostrom et al. indicated that overexpression of irisin via adenoviral vectors in diet-induced obese rodents increases energy expenditure, attenuates weight gain, and improves glucose tolerance compared with control vectors [8]. These initial findings have been corroborated by others via *in vitro* and *in vivo* approaches [9, 10]. In addition, it has been shown that irisin signals via AMP-activated kinase (AMPK) pathway to mediate glucose uptake and fatty acid oxidation in primary myocytes suggesting a putative role in glucose and lipid homeostasis [11]. Together, these discoveries have sparked interest in viewing irisin as a possible anti-obesity therapy [12].

Findings from a recent systematic review [13] and randomized control trial [14] indicates that chronic exercise training does not increase irisin levels and may actually lead to reductions. This parallels the notion that irisin is a metabolic stress-induced chemokine that transiently increases in response to cellular energy demand [15], which is consistent with the secretory pattern of other myokines [7]. Accordingly, a single bout of aerobic and resistance exercise, a known physiologic stimulus that increases whole-body insulin sensitivity (reviewed in [16–22]), reportedly induces a temporary increase in circulating irisin concentrations immediately post-exercise in healthy [5, 23–26] and obese individuals [27], however, these findings are not universal [28, 29] and require replication. Thus, the physiologic relevance of circulating irisin in humans continues to be debated [30–34]. To our knowledge no study has measured irisin levels during exercise in obese females. Given that irisin concentrations are more variable throughout the day in obese compared to lean individuals [35], it is plausible that these individuals may have impaired regulation of irisin during exercise. In addition, it is unknown how time-of-day may affect the irisin response to acute exercise. In this context, the majority of acute exercise studies are carried out following an overnight fast. However, our reasoning to implement afternoon exercise is consistent with the concept that many individuals perform afternoon exercise, whether in the postprandial state or not and thus afternoon exercise may be a better reflection of a real life exercise exposure. Moreover, one prior report indicated that irisin exhibits a diurnal pattern throughout the day [24], which may influence the exercise response.

Thus, the aim of this study was to determine the effect of a single bout of aerobic exercise of differing intensities on circulating irisin concentrations during and post-exercise in obese females. We hypothesized that the greater metabolic stress induced via acute high-intensity interval exercise would enhance peak plasma irisin levels compared with continuous moderate exercise.

## Methods

The Institutional Review Board at the University of Missouri approved this study. A detailed description of the methodology has been previously described [36]. Archived plasma samples from a previous study [36] were analyzed for circulating irisin levels. Sample size was based primary outcome variable of the primary study. Accordingly, eleven young sedentary obese females completed this study. Inclusion criteria included individuals between 18 and 35 years of age, body mass index (BMI) >30kg/m^2^, weight stable over prior 3 months, not regular exercisers, no prior history of lung, kidney, endocrine, or gastrointestinal disease, and not taking any medications known to alter glucose metabolism.

Subjects completed a single bout of exercise or remained sedentary for the duration of the protocol. In a randomized order, intervention protocols consisted of: (1) no exercise (NoEx), (2) moderate-intensity continuous aerobic exercise (ModEx), and (3) high-intensity aerobic interval exercise (IntEx). Interventions were conducted at least 1 week apart until all three were completed as previously described [36]. All subjects were studied between days 1–10 of their menstrual cycle and consumed a standard lunch meal (~1200 h) (~750 kcal, 65%carbohydrate, 20% fat, 15% protein) during each testing day. Both ModEx and IntEx exercise sessions were matched for caloric-expenditure (400 kcal). Participants arrived at the Exercise Physiology Laboratory at approximately 1600 h. During each condition, a venous catheter was inserted for repeated blood sampling at baseline and every 10 minutes thereafter for 190 minutes. ModEx consisted of continuous treadmill walking at 55% VO_2peak_, whereas IntEx was comprised of 4 minutes of high intensity intervals at 80% VO_2peak_ separated by 3 minutes of active recovery (50% VO_2peak_) [36]. Exercise energy expenditure was measured using indirect calorimetry (ParvoMedics’ TrueOne 2400 metabolic cart, Salt Lake City, Utah, UT, USA). Following exercise, participants rested in a recumbent chair for approximately 125 minutes. Plasma irisin levels were determined at t = 0, 30, 50, 80, and 190 minutes, where exercise occurred between t = 10–65 minutes. Peak irisin values occurred at t = 50 min.

Peak oxygen consumption (VO_2peak_) was assessed through indirect calorimetry, using a continuous treadmill protocol [37]. The BOD POD^®^ was used to assess body composition according to the manufacturer instructions (COSMED USA, Concord, CA) [38].

### Blood Analysis

Plasma concentrations of irisin were measured in duplicate using a commercially available enzyme immunoassay kit (EK-067-29, Phoenix Pharmaceuticals, Inc., lot#606666) with a sensitivity of 1.9 ng/ml and range 1.9–1000 ng/ml. The antibody used by this particular kit has been validated against Western blotting [39] and mass spectrometry [9, 10]. Samples were diluted with equal parts sample and assay buffer and fell within the linear range of the standard curve (1.9–26.8 ng/ml). The intra-assay and inter-assay coefficient of variations (CV) were 3.3% and 7.4%, respectively. Since prior evidence indicates that irisin may vary depending on which commercial kits are utilized, we also measured irisin levels with the Adipogen International irisin ELISA kit (Liestal, Switzerland; Cat. #AG-45A-0046EK-KI01), which has a detection range of 1–5000 ng/ml, in order to assess agreement between kits.at baseline (t = 0 min), during exercise (t = 30 min), and post-exercise (t = 80 min). Our reasoning for including another kit is based on concerns raised in a recent review article which cautions against making kit-to-kit comparisons [33]. Glucose and lactate were determined using an YSI SELECT analyzer (Yellow Springs, Ohio). Plasma insulin, C-peptide, and glucagon were analyzed using a MILLIPLEX magnetic bead-based quantitative multiplex immunoassay with the MAGPIX instrumentation (Millipore, Billerica, MA). The intra-assay CV was 5.6%, 7.2% and 4.7% for C-peptide, glucagon and insulin, respectively. The inter-assay CV was 14.7%, 9.8%, and 12.8% for C-peptide, glucagon and insulin, respectively. The homeostatic model of insulin resistance (HOMA-IR) index was used as a surrogate of hepatic insulin resistance [40].

### Statistical Analysis

One-way analysis of variance (ANOVA) with repeated measures was used to assess baseline differences in blood biochemistry and plasma irisin levels. Main effects of time on plasma irisin concentrations were assessed via repeated measures ANOVA. Follow-up post hoc tests with Bonferroni correction (type I error) were run to assess differences in irisin levels from baseline. Peak exercise-induced change in irisin was computed as percent change from baseline (t = 0). Normal distribution of data was determined using Shapiro-Wilk’s test. The assumption of sphericity was tested via Mauchly’s test of sphericity. Pearson r was used to assess associations between irisin and body composition and blood chemistry. All statistical analyses were performed using SPSS statistical software version 20 (IBM Corporation, Armonk, NY, USA). Data are shown as mean ± SEM unless stated otherwise. *P*<0.05 were considered as statistically significant for all analyses.

## Results

**Fig 1:**
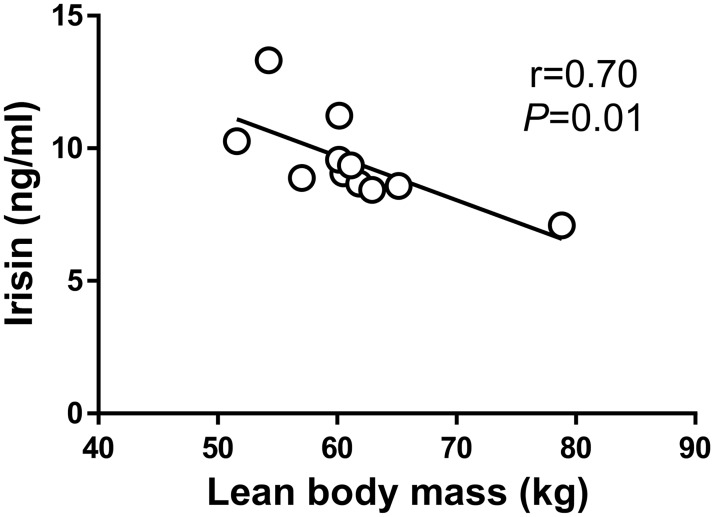
Basal irisin is inversely associated with lean body mass. Correlation was computed via Pearson r.

Table 1 summarizes baseline participant characteristics and blood chemistry. As previously reported [36], fasting glucose, glucagon, and HOMA-IR were not different between study days (*P*>0.05); however, fasting lactate, insulin, and c-peptide were higher during ModEx compared to NoEx study day (*P*<0.05). We did not find significant associations between basal irisin levels and body fat mass, BF%, VO_2_peak, or fasting glucose and circulating hormones [(i.e., insulin, c-peptide, and glucagon), Table 2]. However, lean mass was inversely associated with resting irisin levels (Fig 1, *P*<0.05).

**Table 1:** Participant characteristics and baseline blood chemistry.

Age (years)	24.3 ± 1.4
Height (m)	1.7 ± 0.01
Weight (kg)	107.9 ± 6.7
BMI (kg/m^2^)	37.3 ± 2.1
BF%	42.1 ± 2.5
VO_2_peak (kg/ml/min)	25.2 ± 1.4
Glucose (mg/dl)	74.4 ± 2.1
Insulin (pg/ml)	1090.8 ± 152.7
c-peptide (pg/ml)	2222.3 ± 150.0
Glucagon (pg/ml)	23.1 ± 3.4
Lactate (mg/dl)	6.3 ± 0.5
HOMA-IR	4.9 ± 0.8

n = 11 (female), All values are means ± SEMs. BMI, body mass index; BF%, percent body fat; VO_2_peak, peak oxygen consumption. HOMA-IR, homeostasis model assessment of insulin resistance.

**Table 2:** Associations between basal irisin and anthropometric and metabolic parameters.

	Pearson r	*P* Value
Age (years)	-0.34	0.29
BMI (kg/m^2^)	-0.31	0.34
Fat mass (kg)	-0.10	0.75
BF%	0.17	0.59
VO_2_peak (ml/kg/min)	-0.10	0.75
Glucose (mg/dl)	-0.44	0.17
Insulin (pg/ml)	-0.16	0.63
c-peptide (pg/ml)	-0.47	0.14
Glucagon (pg/ml)	0.08	0.79
Lactate (mg/dl)	-0.27	0.41
HOMA-IR	-0.20	0.54

n = 11 (female), Values are means ± SEMs. BMI, body mass index; BF%, percent body fat; VO_2_peak, peak oxygen consumption. HOMA-IR, homeostasis model assessment of insulin resistance.

**Fig 2:**
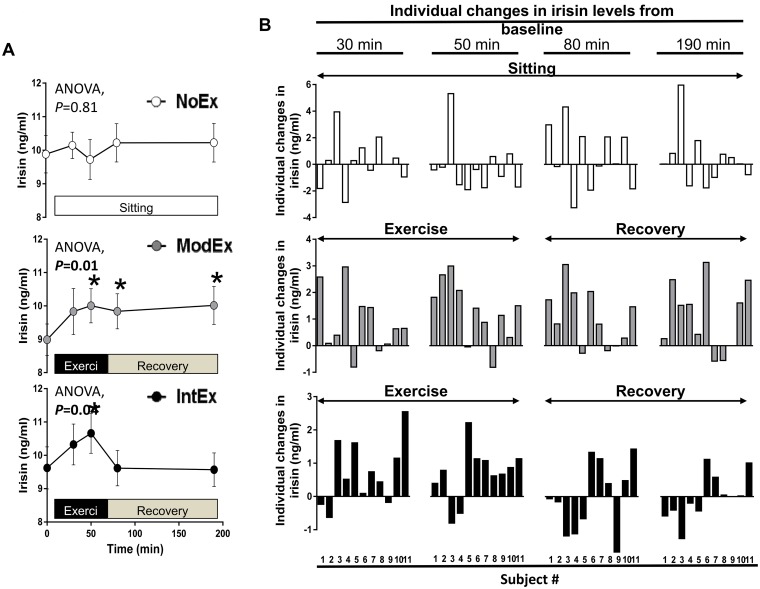
A single bout of moderate and high-intensity exercise increased plasma irisin during exercise. A) irisin curves with or without acute exercise; B) individual irisin responses during NoEx, ModEx, and IntEx. Values are expressed as mean ± SEM. NoEx, no exercise; ModEx, continuous moderate exercise; IntEx, high-intensity interval exercise. *P<0.05 vs baseline.

At baseline, plasma irisin levels were not different among study days (*P* = 0.13) and did not differ across time during NoEx (Fig 2A). However, irisin concentrations were elevated during exercise in both ModEx and IntEx (Fig 2A, *P*<0.05); yet the increase above baseline was short-lived for IntEx, returning to resting levels within 15 minutes post-exercise. In contrast, irisin concentrations remained elevated until the end of testing during ModEx (Fig 2A, *P*<0.05). The peak exercise-induced increase in irisin (i.e., percent increase from t = 0 to t = 50 min) was similar between ModEx and IntEx (*P* = 0.95), increasing by 11.9± 3.4% and 12.3 ± 4.1% (*P*<0.05), respectively. Of note, the absolute increase in peak exercise-induced irisin levels were modest 1.02 ± 0.3 ng/ml and 1.04 ± 0.4 ng/ml during ModEx and IntEx (Fig 2A), respectively. Inconsistent with prior reports [23, 41], plasma irisin levels were not associated with circulating lactate levels which were increased 4.8- and 2.7-fold during IntEx relative to NoEx and ModEx, respectively (*P*<0.001), whereas ModEx resulted in 1.7-fold increase in lactate levels compared to NoEx (*P* = 0.01).

Despite the exercise-induced increase in mean irisin levels, subjects’ responses were variable (Fig 2B). A >5% change (i.e. increase or decrease) in irisin relative to basal value was defined as a significant response. Under this assumption, we found that 68.2% and 63.6% of individuals increased whereas 9.1% and 13.6% decreased plasma irisin concentrations during exercise for ModEx and IntEx, respectively (Fig 2B). On the contrary, during NoEx approximately one-third of subjects increased, decreased, or exhibited no changes in plasma irisin, indicating no predictable pattern (Fig 2B). Notably, 61.8% of subjects’ irisin levels remained elevated during the recovery period of ModEx, whereas 37.8% were elevated following IntEx. It is clear from Fig 2B that multiple individuals responded differently to exercise intensity, where the increase, decrease, or no response in irisin concentrations during ModEx did not consistently match the pattern during IntEx. Moreover, as a whole, it appears that ModEx may produce more predictable irisin responses compared with IntEx.

Given that recent recommendations caution against kit-to-kit comparisons among commercial irisin ELISA kits [33], we compared the EK-067-29 (Phoenix Pharmaceuticals, Inc.) vs AG-45A-0046EK-KI01 (Adipogen International Liestal, Switzerland) ELISA kits to provide context in the same cohort of individuals. Strikingly, we found on average 230-fold higher irisin levels with the Adipogen International irisin ELISA kit (AG-45A-0046EK-KI01) compared to the EK-067-29 (Phoenix Pharmaceuticals, Inc.)(data not shown), thus reinforcing concerns raised by Polyzos and Mantzoros [33].

## Discussion

To our knowledge, this is the first study to report circulating irisin levels during exercise. We found that in young obese women, circulating irisin concentrations are modestly increased during an acute exercise bout; however, in contrast to our hypothesis the peak exercise-induced increase is not augmented during IntEx. In addition, we report what appears to be a dimorphic post-exercise elevation in irisin between ModEx and IntEx, where irisin levels tended to remain elevated >2 hours after ModEx but not IntEx (i.e., return to baseline within 15 min post-exercise). Consistent with the elevation after ModEx, several investigations with healthy individuals indicate that acute exercise stimulates irisin release immediately following exercise [23, 26, 42–44], whereas the exercise-induced irisin response in obese individuals are conflicting [35, 43, 45]. Some of these conflicting reports may be partly attributed to inconsistencies in measurement and reporting of irisin, as previously discussed [30, 33, 46, 47].

The relationship between irisin and adiposity and fat free mass is a matter of debate. Irisin has been identified as a myokine, however, its role in biology extends beyond muscle (i.e., produced by adipose tissue in addition to muscle [48, 49]). Hence, predicting its relationship with descriptive measures of body composition may be difficult. Indeed, we found an inverse association between irisin and lean mass. This finding is consistent with prior data in men and women undergoing strength exercise [50, 51]. However, others indicate a positive association between irisin and BMI, muscle mass (i.e. biceps) and fat free mass [35, 52] and suggest that fat free mass is the primary predictor of irisin levels. Thus, it appears that simple body composition markers may not be consistent indicators of irisin levels.

Few studies have investigated the exercise-induced irisin response during the afternoon. The afternoon period is a time that reflects a general postprandial state (i.e., following breakfast and lunch) in which exercise responses may differ compared to an overnight fast [53]. It is possible that time-of-day effects (i.e. diurnal variations) may influence irisin’s response to acute exercise. In this study baseline irisin concentrations were measured at approximately 1600 h, immediately followed by ~55 minutes of acute exercise or no exercise. Accordingly, we noted that the variation in irisin levels across time was greater during NoEx compared with ModEx and IntEx (Fig 2B). In contrast to NoEx, both ModEx and IntEx increased irisin levels above baseline (11.9± 3.4% and 12.3 ± 4.1%, respectively), suggesting tighter irisin control via exercise. However, the recovery period following ModEx, but not IntEx, appeared to preserve the increase in irisin above basal. Assuming that circulating concentrations of irisin are important for the proposed browning effect on white adipose tissue [8], our data indicate that irisin levels may persist in plasma for longer duration in response to ModEx and therefore may increase the likelihood of irisin-induced adipose tissue browning. This hypothesis is contrary to what has been observed by one study in healthy active individuals, where irisin levels were greater immediately following high-intensity exercise compared to moderate intensity exercise [5]. It has been reported that high-intensity exercise does not increase irisin levels until 6 h post-exercise and remains elevated for an additional 13 h, an effect of which is greater than moderate intensity exercise [42]. The aforementioned studies were conducted with healthy non-obese subjects, the majority of which were men, who may respond differently to high-intensity exercise compared with obese sedentary women.

Herein, we report that peak exercise-induced irisin levels are equally increased by ModEx and IntEx, whereas post-exercise recovery levels remain elevated in ModEx only. However, irisin release may be dependent upon the fitness status of subjects and energy demand of exercise since irisin levels were significantly greater in more fit individuals who exercised longer and reached a higher percentage of VO_2_ max, which was directly associated with lactate levels [23]. In the current study, we did not find an association between irisin concentrations and lactate levels. We speculate that obesity-related impairments in muscle signaling and possibly differences in muscle activation between lean and obese individuals may contribute to the modest elevations in irisin levels during acute exercise and the absence of an ‘intensity effect’. In accordance, Norheim et al. indicated that irisin was non-significantly increased immediately following exercise in individuals with prediabetes, yet, following 12 weeks of exercise training the acute exercise-induced increase in irisin levels reached statistical significance [25]. These data from Norheim et al. support the notion that fitness status of subjects is an important predictor of the exercise-induced irisin response. In contrast, Huh et al. reported that irisin increased in response to acute exercise independent of fitness level in lean men [5]. It is likely that the lack of obesity contributes to these distinctions. Another possibility that should not be overlooked is that acute exercise may increase the autocrine/paracrine effects of various myokines including irisin, driving improvements in muscle function in the absence of robust increases in circulating levels. Consequently, *in vitro* studies have demonstrated that irisin stimulates AMPK activation and thus autocrine/paracrine actions of irisin may modulate exercised-induced changes in skeletal muscle [5], independent of circulating levels.

An intriguing observation of the current study is the individual variability between ModEx and IntEx (Fig 2B). Exercise energy expenditure was held constant so that all subjects expended 400 kcal during exercise. In doing so, we were able to make comparisons between exercise intensity independent of energy expenditure. Together, 65% of individuals significantly increased irisin levels during exercise. However, to our surprise, less than half (40.8%) of these responders exhibited an increase in irisin during both ModEx and IntEx. In fact, 22.6% of the same subjects responded by increasing during one exercise condition and decreasing during the other exercise condition. We also found that of the non-responders only 2 subjects responded in the same direction to ModEx and IntEx. It is possible that exercise intensity initiates distinct differences in the balance between irisin secretion and irisin clearance; yet, this remains to be shown. Accordingly, the implications of these findings are unknown and warrant further investigation.

This study is not without limitations. We did not include a non-obese control group; however, all subjects completed, in random order, all phases of the study and served as their own controls. Subjects were <4 hours postprandial at the onset of exercise. It is unlikely that the prior meal (i.e., standard lunch meal) impacted irisin levels given that several reports have indicated no postprandial influence on circulating irisin levels [24, 35]. In addition to skeletal muscle, irisin is secreted by adipose tissue [48, 49]; hence by sampling plasma levels of this peptide we are unable to determine its origin. Another limitation of the current investigation is that irisin was measured using a commercially available ELISA kit. The validity of several kits have been questioned [32, 54]; with the suggestion that the antibodies may capture cross-reactions with non-specific proteins, introducing a source of variation and even questioning the biologic relevance of irisin. In contrast, some kits have been shown to accurately detect endogenous and exogenous irisin levels in humans [33, 44], one of which was utilized in the current investigation (EK-067-29, Phoenix Pharmaceuticals, Inc.). Accordingly, Jedrychowski et al. unequivocally demonstrated that irisin is present in human plasma using tandem mass spectrometry, which appears to be the goal standard for measurement of the irisin peptide. However, this technique is costly and methodologically tedious. Thus, we utilized the EK-067-29 (Phoenix Pharmaceuticals, Inc.) which has been validated via Western blotting and mass spectrometry [9, 10, 39]. Importantly, caution should be taken when making kit-to-kit comparisons [33]. In accordance with these concerns, we found on average 230-fold higher irisin levels with the Adipogen International irisin ELISA kit (AG-45A-0046EK-KI01) compared to the EK-067-29 (Phoenix Pharmaceuticals, Inc.). Based on these findings, it is reasonable to suggest that using mass spectrometry as the gold standard of measurement may improve future ELISA kits and ultimately enhance our understanding of irisin’s role in physiology.

At present, scientific inquiry regarding irisin is confounded by the lack of a known receptor. Identification of such receptor(s) would open the door to metabolic stress-induced (e.g. exercise, caloric restriction, pharmacotherapy) activation/secretion of the irisin peptide. In this context, it has been suggested that obesity/insulin resistance may promote “irisin resistance”; yet, without a known receptor this hypothesis remains speculative. Nonetheless, given the presumed link between irisin and the regulation of energy homeostasis and insulin sensitivity [12, 55], studies are warranted to identify the mechanisms by which irisin impacts human pathophysiology (e.g., type 2 diabetes).

In summary, these findings indicate that a single bout of moderate and high intensity afternoon exercise induces modest increases in circulating irisin during exercise; however the regulation post-exercise appears to be dimorphic between exercise intensity in obese females. Additional research is needed to elucidate the potential interaction between exercise intensity and time-of-day effects on autocrine/paracrine and endocrine role of exercise-induced irisin secretion in obese humans.

## Supporting Information

S1 Table Study data. (XLSX)

## Abbreviations

IntEx: interval exercise
ModEx: moderate exercise
NoEx: no exercise
PGC1α: peroxisome proliferator-activated receptor-γ coactivator-1 α
